# Myoelectric Activity of the Peroneal Muscles Following Lateral Ankle Sprain: A Cross-Sectional Analysis

**DOI:** 10.3390/jfmk10020179

**Published:** 2025-05-15

**Authors:** Oriol Casasayas-Cos, Noé Labata-Lezaun, Albert Pérez-Bellmunt, Carlos López-de-Celis, Johke Smit, Xavier Marimon-Serra, Ramón Aiguadé-Aiguadé, Joaquín Sanahuja-Diez-Caballero, Max Canet-Vintró, Luis Llurda-Almuzara

**Affiliations:** 1 Faculty of Medicine and Health Sciences, Universitat Internacional de Catalunya, 08195 Barcelona, Spain; casasayascos@uic.es (O.C.-C.); carlesldc@uic.es (C.L.-d.-C.); maxcanet44@uic.es (M.C.-V.); 2 Actium Functional Anatomy Research Group, 08195 Sant Cugat del Vallès, Spain; 3 Centre Cos Morera-Fisioterapia Osteopatia, 08225 Terrassa, Spain; 4 Facultad de Salud, Universidad Vitoria-Gasteiz (EUNEIZ), La Biosfera Ibilbidea, 6, 01013 Vitoria-Gasteiz, Spain; 5 PhASRec, Faculty of Health Sciences, North-West University (NWU), Potchefstroom 2520, South Africa; 6 Department of Strength of Materials and Structural Engineering, Universitat Politècnica de Catalunya (UPC-Barcelona TECH), 08028 Barcelona, Spain; 7 Institut de Recerca Sant Joan de Deu (IRSJD), 08950 Barcelona, Spain; 8 Nursing and Physiotherapy Department, University of Lleida, 25003 Lleida, Spain

**Keywords:** ankle, ankle injuries, electromyography, athletes, sprain, muscle contraction

## Abstract

**Background**: Lateral ankle sprains can result in adverse outcomes, including reinjuries or chronic ankle instability. The peroneal musculature plays a key role in stabilizing the ankle and preventing sudden ankle inversions that may lead to sprains. **Objective**: The purpose of the study is to investigate (1) inter-limb differences in peroneal myoelectrical activity in athletes with a history of ankle sprain during the past six months and (2) to investigate peroneal myoelectrical activity differences between athletes with and without a history of ankle sprain. **Methods**: Sixty-seven athletes (53% females, 46.3% males) were included in this observational cross-sectional study. Self-reported data regarding history of ankle sprain were collected. The peroneal myoelectrical activity was obtained during (1) isometric ankle eversion, (2) dynamic ankle eversions, (3) single leg squat, (4) unilateral and (5) bilateral drop jump test, (6) sprint, and (7) change of direction. **Results**: No significant differences in peroneal myoelectrical activity were observed between individuals with (n = 46) and without (n = 21) a history of ankle sprain in the past six months (*p* > 0.05). Additionally, no significant inter-limb differences were found within the previous ankle sprain group (*p* > 0.05). **Conclusions**: This study found no significant inter-limb differences in peroneal muscle activity among athletes with a history of ankle sprain during the past six months. Moreover, no differences were observed between athletes with and without a history of ankle sprain. This study has certain limitations, including the lack of data regarding the timing and severity of the ankle sprain, as well as the duration and specific characteristics of the rehabilitation process.

## 1. Introduction

An ankle sprain is one of the most common injuries worldwide and the most prevalent musculoskeletal injury in the physically active population [1,2]. The prevalence of this injury accounts for 3–5% of all cases presenting to the Emergency Departments in the United Kingdom [3] and has an incidence rate of 3.26 per 1000 person-years in the United States [4]. Furthermore, half of all ankle sprain injuries are sport-related, accounting for 30% of all sport-related injuries [4,5,6]. A considerable body of research and literature is available on ankle sprain rehabilitation. However, although knowledge in the field is improving, injury rates have remained high over the past years [3,5,7]. One of the most prevalent side effects of an ankle sprain is chronic ankle instability (CAI) [1,8,9]. The main risk factor for suffering from an ankle sprain is to have a previous history of this type of injury [1]. CAI is typically characterized by recurrent sprains, ankle collapses, and ankle functional deficits, which may persist following the initial injury [10]. Peroneal musculature function is crucial for providing stability to the ankle [11]. It especially protects ankle lateral ligaments from rapid, uncontrolled, and beyond-the-physiological-range-of-motion inversion and internal rotation of the foot, which has been described as the main mechanism for ankle sprain injuries [12,13]. Therefore, the main objective in rehabilitation is to recover the motor control and neuromuscular response of this musculature to prevent reinjuries or CAI [10,14]. Neuromuscular response (NMR) and motor control are the result of the coordinated action between the muscular and nervous systems to produce a certain mechanical work [15,16]. The mechanical function of the peroneal musculature is to provide ankle stability by preventing rapid inversion, thereby reducing the risk of an ankle sprain.

Surface electromyography (sEMG) is a valid tool for conducting this assessment [17,18]. It has been widely used to detect NMR differences between individuals with or without previous hamstring strains and other musculoskeletal injuries [19]. Research suggests that deficits in the myoelectrical activity of the peroneal musculature may lead to adverse outcomes, including reinjury and the development of CAI [20,21]. While some studies have investigated differences in sEMG activity of the peroneal musculature between limbs and among athletes following an ankle sprain or those with CAI [22,23,24], only Allet et al. [22] assessed this during a dynamic test using a single-hop test. However, no studies have evaluated peroneal sEMG responses during dynamic tests that incorporate changes of direction, varied jump styles, and conditions of instability, movements that closely replicate the mechanism of injury, such as plantar flexion, adduction, and inversion [25].

Thus, the purpose of this study is (1) to analyze peroneal muscle activity differences between injured and non-injured limbs in athletes with a history of ankle sprain during the past six months during dynamic sport maneuvers and (2) to analyze peroneal muscle activity differences between athletes with and without a history of an ankle sprain in dynamic sport maneuvers. This information could greatly benefit clinicians in their efforts to improve the treatment and prevention of ankle sprains.

## 2. Materials and Methods

### 2.1. Design and Participants

An observational cross-sectional study was carried out, following the STROBE reporting guidelines (Strengthening the Reporting of Observational Study) statement for observational studies [26]. The study was performed in the functional anatomy laboratory of the Universitat Internacional de Catalunya (UIC-BCN). The sample size was calculated using G*Power v3.1.9.7, based on the root mean square (RMS) of the peroneal muscles’ electromyographic activity, expressed as a percentage of Maximal Voluntary Isometric Contraction (%MVIC). Assuming a large effect size (Cohen’s d = 0.80), as reported in the previous health literature [12], and using a two-tailed independent samples *t*-test with an alpha level of 0.05 and a statistical power of 80%, a minimum of 60 participants was required. To account for an estimated 10% dropout rate, the final target sample size was set at 66 participants, distributed in a 2:1 ratio (45 with a history of lateral ankle sprain and 21 without), reflecting the cumulative prevalence of ankle sprains in athletic populations [1,3]. Participants were recruited via flyers posted on noticeboards in common areas at the university and club facilities.

To be included in the study, athletes had to (1) be completely engaged (in perfect health) in the upcoming training session of their sport; (2) exercise >6 h per week to improve performance [27]; and (3) have suffered a single lateral ankle sprain during the past six months diagnosed by medical staff. To classify participants within this group, the inclusion criteria were based on the International Ankle Consortium criteria [11]. Accordingly, the patient or their clinical history had to indicate the following: (1) a history of an acute traumatic event involving an inversion mechanism of the ankle, often occurring during weight-bearing activities such as walking, running, or jumping; (2) pain localized over the anterolateral aspect of the ankle, particularly around the anterior talofibular ligament (ATFL) and/or the calcaneofibular ligament (CFL); (3) swelling and/or ecchymosis (bruising) in the lateral ankle region, developing within the first 24–48 h after injury; (4) tenderness upon palpation over the ATFL, CFL, or other lateral ligamentous structures; (5) positive special tests such as the anterior drawer test or the talar tilt test, indicating ligamentous laxity; (6) limited weight-bearing ability and/or reduced range of motion, especially during plantarflexion and inversion; (7) no evidence of fracture. A single ankle sprain was sufficient to be included in the “Ankle Sprain Group” because it has been previously associated with future risk of ankle sprain, and other studies have used similar criteria [28,29]. Athletes were excluded from the study if (1) they were unable to perform the test because of an injury or illness; (2) were unable to understand English, French, or Spanish; and (3) had previous history of surgery due to an ankle injury.

Before agreeing to participate, all athletes were informed about the procedure and purpose of the study, and they gave written informed consent to participate. This study (Ref. number CBAS201914, 15 November 2019) was approved by the ethical committee at the Universitat Internacional de Catalunya, ensuring that the research was conducted ethically and responsibly and that the rights and welfare of all participants were protected throughout the study.

### 2.2. Testing Procedure

Participants in the study were asked to provide their personal information (age, weight, height, days of training per week, dominancy) and history of ankle sprain (limb affected, medical staff who diagnosed the sprain, other lower limb injuries). If the athlete had suffered an ankle sprain diagnosed by medical staff during the past six months, they were classified into the “Ankle Sprain Group”.

**Figure 1:**
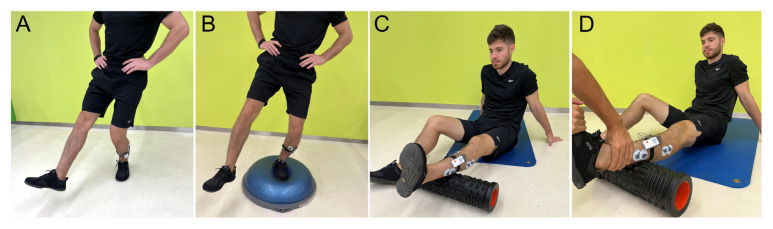
(**A**) Single leg squat, (**B**) BOSU ball, (**C**) dynamic ankle eversions, (**D**) Maximal Voluntary Isometric Contraction (MVIC).

**Figure 2:**
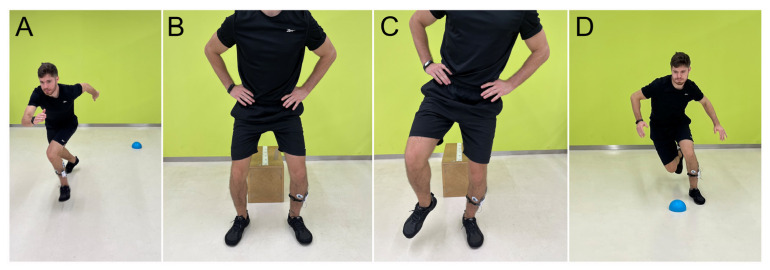
(**A**) Sprint, (**B**) bilateral drop jump, (**C**) unilateral drop jump, (**D**) change of direction.

Most of the tests consisted of dynamic sports maneuvers designed to replicate potential mechanisms. The evaluated movements included the following: (1) Maximal Voluntary Isometric Contraction (MVIC) of ankle eversion, in which the participant performed a maximal ankle eversion against manual resistance for 5 s; (2) dynamic eversions, during which participants executed five dynamic ankle eversions without resistance; (3) single leg squat (SLS), requiring the participant to squat as deep as possible with one leg while keeping their hands on the hips; (4) unilateral (UDJ) and (5) bilateral (BDJ) drop jump tests, in which the participant landed from a 30 cm box with one leg and two legs, respectively; (6) the both sides up (BOSU) ball stability exercise, in which participants had to maintain balance on the BOSU ball with one leg for five seconds; (7) sprint, where athletes had to sprint a 10 m distance; and (8) change of direction exercise, during which athletes had to run 10 m and then change the direction 45° toward the opposite leg being assessed. The randomization sequence of the tests was performed using the software www.random.org to avoid possible biases in the measurements. Only one attempt was measured, due to the number of tests evaluated. However, for familiarization purposes, for exercises 3, 4, 5, and 6, participants were allowed to perform up to two practice attempts without measurement. Figure 1 and Figure 2 show how tests were performed.

All tests, except the change of direction, started with an acoustic signal and ended when the participant completed the test. In the eversion tests, the trial ended when the participant completed the fifth movement. During squats, it ended when the participant returned to their initial position. In drop jumps, the test ended when the participant stabilized. The BOSU test ended five seconds after the acoustic signal. The sprints concluded when the participant crossed the 10 m line. The change of direction test started at the penultimate contact before the change of direction, and it ended when the participant lifted their foot at the final contact of the leg involved in the maneuver.

Ag/AgCl electrodes for surface electromyography electrodes were placed following the SENIAM project recommendations [30] on the peroneus longus and brevis muscles of one limb. The myoelectrical signal was recorded with the mDurance amplifier (mDurance Solutions SL, Granada, Spain) with a sampling frequency of 1024 Hz, providing high-resolution EMG data for analysis. The recorded data were transmitted via Bluetooth to a host computer, which was processed using mDurance^®^ software (mDurance Solutions SL, Granada, Spain) (https://mdurance.com/en/ accessed on 12 November 2024). The amplifier device was positioned on the lateral aspect of the leg to ensure accurate recording of the peroneal muscle activity. The recorded EMG data were preprocessed using a moving average filter. The filter was applied with a window size of 250 ms and a 50% overlap, which helped to reduce noise and improve the signal-to-noise ratio of the data. Before testing, participants were asked to perform a five-minute warm-up targeting active joint mobility. ThaBefore the commencement of the test, the principal researcher gave a demonstration of each test.

Maximal Voluntary Isometric Contraction (MVIC) during an ankle eversion contraction test for 5 s was used to normalize the data. The calculation of the MVIC involves determining the peak of the “root mean square” (RMS) signal obtained during the isometric test. Thus, all data were normalized by ankle eversion MVIC raw signal. The principal variable recorded for myoelectrical activity was RMS activation, expressed as %MVIC. Moreover, maximal activation, expressed as %MVIC, and distribution between peroneus longus and peroneus brevis were also recorded. EMG bursts were assessed from the beginning to the finish of the test, as described in the testing procedure section.

### 2.3. Statistical Analysis

Statistical analysis was conducted using SPSS software (version 26.0, IBM Corp, Armonk, NY, USA). For descriptive statistics, means and standard deviation (SD) were used for continuous variables, and numbers and percentages for categorical variables. Normal distributions of all variables were tested using the Shapiro–Wilk test. Two different comparisons were carried out in the study. The first comparison involved the “Ankle Sprain Group”, in which the previously injured limb was compared to the uninjured limb versus the uninjured lateral limb. The second comparison was between the “Ankle Sprain Group” and the “Control Group”; the previously injured limb was compared to the dominant limb of individuals without previous ankle injuries. Differences between previously injured limbs and non-previously injured limbs from the same participant (inter-limb differences) were tested using a paired *t*-test or a Wilcoxon test, depending on the normality of the distribution. Additionally, differences between previously injured participants’ limbs and non-previously injured participants’ dominant legs (differences between groups) were tested using an unpaired *t*-test or a Mann–Whitney U test, depending on the normality of the distribution. Effect sizes were calculated using Cohen’s d coefficient [31]. An effect size of >0.8 was considered large; around 0.5, intermediate; and <0.2, small [27]. The level of statistical significance was set at *p* = 0.05.

## 3. Results

**Figure 3:**
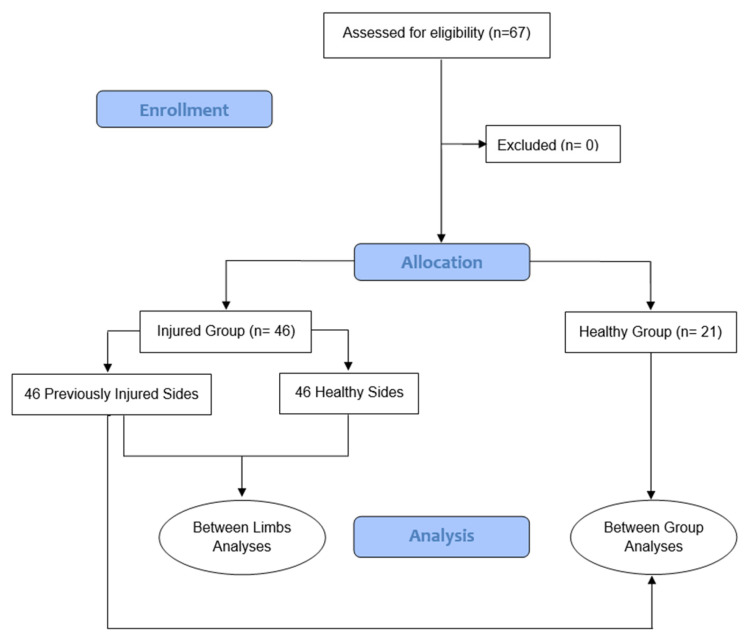
Flow chart. Participants were classified into the “Control Group” and “Ankle Sprain Group” according to whether they had previous ankle injuries. In the Ankle Sprain Group, both the injured limb (Previously Injured Side) and the non-injured limb (Control Group) were analyzed for comparison.

We recruited sixty-seven individuals to participate in this cross-sectional study (Figure 3).

The demographic characteristics are presented in Table 1. The sample group consisted of thirty-six females (53.7%) and thirty-one males (46.3%). Seventy-nine percent (79%) of participants were right-legged, and twenty-one percent (21%) were left-legged. The sport most common in the sample group of this study was soccer (40%), followed by hockey (22%) and fitness (17%).

Twenty-one participants (31%) declared not having sustained an ankle sprain during the past six months and were assigned to the “Control Group”. On the other hand, forty-six participants (69%) indicated having sustained an ankle sprain within the past six months, which was diagnosed by medical staff, and were therefore assigned to the “Ankle Sprain Group”. The Mann–Whitney test revealed no significant baseline differences between groups, as expressed in Table 1, with a low to moderate effect size.

As outlined in the Methods section, two separate statistical analyses were performed. First, differences between the legs of the “Ankle Sprain Group” were evaluated to identify differences between the previously injured leg and the healthy leg of the same participant. All variables in this analysis were tested using the Wilcoxon test, except for “MVIC peroneus longus RMS”, which was tested by a paired *t*-test. Table 2 shows no significant differences (*p* > 0.05) were found for any of the eight dynamic tests, with a low to moderate effect size. Furthermore, no significant differences regarding myoelectric activity distribution and maximal myoelectric activity between the peroneus longus and brevis muscles were found.

Although no significant differences were found between the previously injured side and the healthy side, it is important to note that ES for the peroneus brevis muscle was between 0.21 and 0.38, in contrast to effect size (ES) for the peroneus longus muscle, which ranged from 0.03 to 0.17. This suggests that the peroneus brevis muscle may be more influenced and/or affected by the presence of a previous ankle sprain compared to the peroneus longus muscle. In fact, for all dynamic sports maneuvers, peroneus longus had lower mean values of muscle activity on the previously injured side compared to the healthy side.

Secondly, this study aimed to assess myoelectric activity differences between participants in the “Control Group” and those in the “Ankle Sprain Group”. For this analysis, the injured leg from the “Ankle Sprain Group” and the dominant leg from the “Control Group” were selected. Table 3 shows no significant differences (*p* > 0.05) for any of the eight tests found, with low to moderate effect sizes. Variables from this analysis were tested by unpaired *t*-test, but “MVIC peroneus longus RMS” and “MVIC peroneus brevis RMS” were tested by the Mann–Whitney U test. No significant differences regarding myoelectric activity distribution between the peroneus longus and brevis muscles or maximal myoelectric activity were observed. Moreover, by analyzing mean values and ES, the differences between the two groups can be considered negligible or inconsequential.

## 4. Discussion

The results of this analysis indicated no significant differences in peroneal muscle activation between the previously injured and uninjured legs. This finding suggests that following a single ankle sprain, the injured leg may have fully recovered its myoelectric activity, returning to levels comparable to the uninjured leg. Additionally, the study found no differences in muscle activation levels between athletes with a history of ankle sprains and those without such a history in the past six months. These results suggest that a single ankle sprain may not be sufficient to detect significant differences in peroneal muscle activation between the injured and uninjured limbs. This finding opens possibilities for future research to assess whether such differences exist in individuals with multiple sprains or CAI.

The present results align with those of Rodrigues et al. [24], who found no differences in the contraction intensity of the peroneus longus and brevis muscles between subjects with and without a history of ankle sprain during an injury simulation. Donnelly et al. [23] also reported no sEMG amplitude differences for peroneal muscles during an isometric contraction between patients with and without CAI. Allet et al. [22] found no differences in peroneal muscle activation between healthy and previously injured patients recorded four weeks post-injury during a single hop test.

Contrary to previous studies, this research aimed to incorporate more dynamic tests and movement patterns that closely resemble injury mechanisms such as single-leg and double-leg drop jumps, single-leg squats, sprints, change of direction, or ball training [11,28,29]. The purpose was to reproduce situations where the athlete must have optimal ankle motor control to prevent a rapid and uncontrolled ankle inversion, and subsequently, an ankle sprain. However, as shown in the results, we did not find changes in peroneal muscle activity in any of the tests.

Participants were athletes who trained a minimum of three times per week (a minim of 4 h per week) and were currently available for upcoming training sessions, matches, or competitions. All subjects from the “Ankle Sprain Group” had fully recovered from their ankle sprain following a rehabilitation program. Results from this study suggest that myoelectric activity from the peroneus longus and brevis muscles does not differ from subjects without previous ankle injuries.

Two studies from Kazemi et al. [20] and Jian-Zhi et al. [21] showed myoelectric activity deficits for the peroneus longus muscle in subjects with CAI. However, Koldenhoven et al. [32] found higher myoelectric activity for the peroneus longus muscle during walking in young adults with chronic ankle instability. CAI is a chronic condition resulting from recurrent ankle sprains, leading to a loss of control over the ankle joint. However, this condition was not assured within our “Ankle Sprain Group”, as the inclusion criteria required participants to have sustained a single ankle sprain within the past six months. Thus, the results of this study suggest that changes in myoelectric activity of the peroneal musculature could be present in subjects with CAI, but not in those with a history of a single ankle sprain.

An additional consideration is the possibility of bilateral neuromuscular alterations following a unilateral ankle sprain because of central sensorimotor reorganization. Previous research has demonstrated that individuals with CAI may exhibit altered postural control not only on the injured limb but also on the contralateral, seemingly “healthy” limb. Deodato et al. identified postural control deficits during single-leg stance tasks using inertial sensors, even in the non-injured limb of individuals with CAI, suggesting systemic adaptations beyond the site of injury [33]. Similarly, a systematic review and meta-analysis by Han et al. concluded that proprioceptive deficits are a hallmark feature of CAI and may affect both limbs due to neuroplastic changes within the central nervous system [34]. These findings contrast with the present results, which did not show significant differences in peroneal muscle activation between the injured and uninjured limbs following a single sprain. This may reflect a lower degree of central adaptation in individuals without recurrent instability. Future studies should further explore whether unilateral injuries can induce bilateral neuromuscular impairments, particularly during dynamic and high-demand motor tasks.

An important and highly prevalent musculoskeletal sports injury such as a hamstring strain has been evaluated with the same purpose within the literature. Areia et al. [19] demonstrated a significant decrease in biceps femoris myoelectric activity six months after the return to play. However, they did not specify the maximum or minimum time frame to classify a subject as having sustained a previous hamstring injury. Sole et al. [35] also demonstrated decreased sEMG activation in athletes who had sustained a hamstring injury from 6 weeks to 12 months prior to the study. However, the nature of a muscle injury, as a hamstring strain, differs a lot from a joint injury like an ankle sprain, which may help explain the difference between their findings and those of our study.

This study measured myoelectric activity but no other components of neuromuscular control, such as strength, tone, or stiffness of the peroneal muscles. Some authors [23,36,37,38,39] have already demonstrated ankle eversion strength deficits in athletes with CAI. Perron et al. [40] found significant, though small, deficits in peroneus muscle strength in subjects with an ankle sprain occurring between 8 weeks and 6 months prior to testing. However, we did not find studies assessing peroneus muscle tone or stiffness in previously injured subjects. Therefore, future research should focus on evaluating persistent changes after an ankle sprain in different components of neuromuscular response such as tone or stiffness.

The findings of the present study provide valuable information on the effects of ankle sprain injuries on myoelectrical activity and could serve as a basis for the development of rehabilitation strategies for individuals with a history of ankle sprains. In most cases, a single ankle injury is unlikely to significantly alter the myoelectrical activity of the peroneal musculature. Therefore, without prior analysis, incorporating exercises to improve peroneal muscle activation indiscriminately should not be a primary focus in the rehabilitation of ankle sprains.

Regarding future research directions, studies should focus on investigating other neuromuscular factors such as activation timing, muscle tone, muscle strength, and joint stiffness. Additionally, investigating the relationship between myoelectric activity, the severity of ankle sprains, and time since injury could provide further insights into the long-term effects of ankle sprains on neuromuscular function. This information could help develop individualized rehabilitation strategies for people with a history of ankle sprains.

Taken together, our findings support the hypothesis that a single, adequately rehabilitated lateral ankle sprain may not be sufficient to induce persistent neuromuscular deficits in peroneal muscle activation, unlike cases involving chronic ankle instability (CAI) or recurrent sprains. We therefore hypothesize that the presence, magnitude, and chronicity of neuromuscular alterations are not solely determined by the occurrence of injury but are influenced by additional factors such as injury recurrence, rehabilitation quality, and central sensorimotor integration.

This study has several important limitations. First, the time elapsed since the ankle sprain was not recorded, preventing us from determining when the myoelectric activity of the peroneus longus and brevis muscles may have recovered. As a result, potential differences between individuals injured two months ago versus six months ago could not be assessed. Second, the severity of the sprain was not documented. Given that more severe sprains are associated with greater tissue damage and longer recovery times, this lack of information limits our ability to interpret the extent of neuromuscular impairment across participants. Third, neither the type nor the duration of rehabilitation received was known, limiting our ability to account for their influence on neuromuscular recovery. These factors are critical and could have significantly impacted the observed outcomes.

Furthermore, the study focused exclusively on peroneal muscle activation. While these muscles play a key role in ankle eversion, the potential contribution of surrounding muscle groups through coactivation was not assessed and may have influenced the findings. Lastly, due to the observational nature of the study, causal relationships cannot be established. Future interventional studies are needed to determine whether targeted strategies aimed at enhancing peroneal muscle function can reduce the risk of recurrent ankle sprains.

## 5. Conclusions

In conclusion, this study examined the myoelectric activity of the peroneus longus and brevis muscles in athletes who had sustained a single ankle sprain in the past six months. The results suggest that there is no significant difference in the myoelectric activity of the peroneal muscles between athletes with and without a history of ankle sprain in the last six months. Furthermore, no significant difference was observed in the myoelectric activity of the peroneal muscles between the previously injured limb and the uninjured limb in athletes who had experienced an ankle injury in the last six months.

## Figures and Tables

**Table 1:** Baseline characteristics.

	Control Group (n = 21)	Ankle Sprain Group (n = 46)	
	Mean ± SD or n (%)	Mean ± SD or n (%)	*p*-Value
Gender			
Female	10 (47.6%)	26 (56.5%)	0.498 ^Ŧ^
Male	11 (52.4%)	20 (43.5%)	
Age	20.81 ± 3.74	21.28 ± 4.31	0.795 *
Height	173.14 ± 6.88	172.59 ± 9.48	0.850 *
Weight	64.57 ± 8.92	66.33 ± 12.73	0.556 *
BMI	21.46 ± 1.81	22.17 ± 3.09	0.503 *
Dominance			
Left	4 (19%)	10 (21.7%)	0.802 ^Ŧ^
Right	17 (81%)	36 (78.3%)	
Sport			
Basketball	1 (5.6%)	6 (11.4%)	
Fitness	6 (33.3%)	6 (11.4%)	
Soccer	5 (22.2%)	22 (50%)	0.013 ^Ŧ Ŧ^
Hockey	4 (16.7%)	11 (25%)	
Others	5 (22.2%)	1 (2.3%)	

Abbreviations: SD: Standard Deviation, n: Number, %: Percentage; *p*-value: ^Ŧ^ Chi Square; ^Ŧ Ŧ^ Fisher’s exact test; * Mann–Whitney U test.

**Table 2:** Differences between sides.

	Previously Injured Side Mean ± SD (n = 46)	Control Side Mean ± SD (n = 46)	ES	*p*-Value
Maximal Voluntary Isometric Contraction PB RMS	271.01 ± 147.59	269.09 ± 192.90	0.01	0.746 *
Maximal Voluntary Isometric Contraction PL RMS	204.10 ± 107.57	192.91 ± 92.56	0.11	0.522 **
Dynamic Eversions PB RMS	19.09 ± 12.08	23.49 ± 16.72	0.30	0.192 *
Dynamic Eversions PL RMS	26.70 ± 34.27	27.91 ± 21.52	0.04	0.220 *
Single Leg Squat PB RMS	31.99 ± 22.07	37.86 ± 33.18	0.21	0.912 *
Single Leg Squat PL RMS	61.57 ± 97.43	55.82 ± 42.47	0.08	0.975 *
Drop Jump PB RMS	12.61 ± 7.70	17.16 ± 19.69	0.30	0.667 *
Drop Jump PL RMS	20.11 ± 19.65	19.40 ± 16.26	0.04	0.868 *
Unilateral Drop Jump PB RMS	24.90 ± 14.76	36.96 ± 49.65	0.33	0.294 *
Unilateral Drop Jump PL RMS	39.26 ± 34.19	37.39 ± 20.55	0.07	0.792 *
Bosu PB RMS	48.74 ± 22.19	60.48 ± 42.38	0.35	0.103 *
Bosu PL RMS	71.28 ± 66.07	73.17 ± 53.27	0.03	0.935 *
Sprint PB RMS	78.08 ± 39.03	94.90 ± 72.00	0.29	0.411 *
Sprint PL RMS	128.54 ± 112.30	119.71 ± 107.64	0.08	0.483 *
Change of Direction PB RMS	76.68 ± 44.38	100.82 ± 79.22	0.38	0.115 *
Change of Direction PL RMS	146.29 ± 173.23	122.34 ± 90.58	0.17	0.769 *

Abbreviation: * Wilcoxon test; ** Paired *t*-test; PB: Peroneus brevis; PL: Peroneus Longus; RMS: Root Mean Square; SD: Standard Deviation; ES: Cohen’s d.

**Table 3:** Differences between groups.

	Injured Group Mean ± SD (n = 46)	Healthy Group Mean ± SD (n = 21)	ES	*p*-Value
Maximal Voluntary Isometric Contraction PB RMS	249.13 ± 108.23	227.35 ± 93.09	0.22	0.434 *
Maximal Voluntary Isometric Contraction PL RMS	191.05 ± 60.04	186.93 ± 106.05	0.05	0.875 *
Dynamic Eversions PB RMS	25.64 ± 18.63	21.78 ± 16.28	0.22	0.298 **
Dynamic Eversions PL RMS	28.64 ± 16.62	29.69 ± 34.64	0.04	0.226 **
Single Leg Squat PB RMS	36.65 ± 23.49	36.44 ± 33.46	0.01	0.844 **
Single Leg Squat PL RMS	47.82 ± 30.54	50.81 ± 38.47	0.09	0.933 **
Drop Jump PB RMS	13.87 ± 13.86	12.42 ± 7.17	0.13	0.508 **
Drop Jump PL RMS	16.90 ± 17.24	19.78 ± 17.93	0.16	0.234 **
Unilateral Drop Jump PB RMS	27.14 ± 24.95	26.90 ± 16.76	0.01	0.525 **
Unilateral Drop Jump PL RMS	35.85 ± 35.62	39.27 ± 32.38	0.10	0.579 **
Bossu PB RMS	46.57 ± 23.18	51.90 ± 28.83	0.20	0.582 **
Bossu PL RMS	51.92 ± 19.97	73.40 ± 66.42	0.44	0.436 **
Sprint PB RMS	76.36 ± 60.66	74.31 ± 38.59	0.04	0.480 **
Sprint PL RMS	81.82 ± 44.36	106.13 ± 69.48	0.42	0.477 **
Change of Direction PB RMS	82.87 ± 61.08	74.37 ± 49.50	0.15	0.749 **
Change of Direction PL RMS	92.04 ± 55.85	98.39 ± 75.59	0.10	0.848 **

Abbreviation: * Mann–Whitney U; ** Unpaired *t*-test; PB: Peroneus brevis; PL: Peroneus Longus; RMS: Root Mean Square; SD: Standard Deviation; ES: Cohen’s d.

## Data Availability

The data presented in this study are available on request from the corresponding author.
